# Nurses' Practice Environment and Work-Family Conflict in Relation to Burn Out: A Multilevel Modelling Approach

**DOI:** 10.1371/journal.pone.0096991

**Published:** 2014-05-12

**Authors:** Constanze Leineweber, Hugo Westerlund, Holendro Singh Chungkham, Rikard Lindqvist, Sara Runesdotter, Carol Tishelman

**Affiliations:** 1 Stress Research Institute, Stockholm University, Stockholm, Sweden; 2 Karolinska Institutet, Department of Learning, Informatics, Management and Ethics, Medical Management Center, Stockholm, Sweden; Universität Bochum, Germany

## Abstract

**Objectives:**

To investigate associations between nurse work practice environment measured at department level and individual level work-family conflict on burnout, measured as emotional exhaustion, depersonalization and personal accomplishment among Swedish RNs.

**Methods:**

A multilevel model was fit with the individual RN at the 1st, and the hospital department at the 2nd level using cross-sectional RN survey data from the Swedish part of RN4CAST, an EU 7th framework project. The data analysed here is based on a national sample of 8,620 RNs from 369 departments in 53 hospitals.

**Results:**

Generally, RNs reported high values of personal accomplishment and lower values of emotional exhaustion and depersonalization. High work-family conflict increased the risk for emotional exhaustion, but for neither depersonalization nor personal accomplishment. On department level adequate staffing and good leadership and support for nurses reduced the risk for emotional exhaustion and depersonalization. Personal accomplishment was statistically significantly related to staff adequacy.

**Conclusions:**

The findings suggest that adequate staffing, good leadership, and support for nurses are crucial for RNs' mental health. Our findings also highlight the importance of hospital managers developing policies and practices to facilitate the successful combination of work with private life for employees.

## Introduction

Burnout is a commonly studied outcome and well-recognized problem among health care staff which can lead to ill-health for the individual [1], poorer quality of care for patients [2], and occupational attrition [3], [4] with related expenses. According to Maslach, it is a syndrome that can occur among individuals who work with people in some capacity [5], with three dimensions of burnout differentiated, namely emotional exhaustion, depersonalization, and personal accomplishment. Emotional exhaustion, conceptualized as the most central component of burnout, refers to a feeling of being overextended and depleted of one's emotional and physical resources; depersonalization describes negative, callous, or excessively detached responses to various aspects of the job; whereas diminished personal accomplishment includes feelings of incompetence and a lack of achievement and productivity in work [6]. As mentioned above, burnout has been related to many severe outcomes for health staff and patients; e.g. Aiken et al. 's seminal research [7] pointed to the relationships between registered nurses' (RN) burnout and patient outcomes, with higher patient-RN ratios related to both increased mortality for patients and increased risk for RN burnout and job dissatisfaction. These relationships have been supported by later research [8]–[11]; a large body of literature has described the influence of modifiable dimensions of nurses' work environment and workload on burnout rates [12]–[14]. A meta-analysis of burnout revealed that perception of job demands, resources, and organisational attitudes were significantly related to all three aspects of burnout [15].

Even the relationship between work-family conflict and burnout has been well established in previous research showing a consistent effect of work-family conflict on emotional exhaustion and burnout [16], [17]. Indeed, one study identified work-family conflict as one of the best predictors of emotional exhaustion and depersonalization [18]. Many characteristics of work situations common among RNs, e.g. shift work, long working hours and responsibility for others, have been identified as increasing risk for work- family conflict [19]-[21]. However, research examining work-family conflict within health professions is generally rather rare [22], although the limited empirical data shows rather high proportions of work-family conflict among nurses [23].

Most decisions affecting the work practice environment for nursing care are made at different organisational levels (e.g. nursing unit, department or hospital). While some work practice environment research has considered the shared experiences of nurses in particular units or hospitals, many studies have been restricted to consideration of correlations between individual nurses' ratings of their workplace [4]. However, if problem areas are to be addressed, it is important to tease apart how factors on different organisational levels may relate to RN burnout. As Bowers et al. [24] recognized there is a need to address staff issues at every organisational level. To date, only a few studies have examined issues at different organisational levels. Van Bogaert et al. [25] explored the nursing unit level relationships between nurse work environment dimensions and burnout using a 2-level linear mixed model for each of the three burnout dimensions separately and found significant relationships for all environment coefficients. More recently Li et al. [26] investigated the group-level impact of nurse work environment dimensions on burnout, adapting a series of multilevel statistical models incorporating four different levels (country, hospital, nursing unit, and nurse). They found that nurse work environment dynamics are related to nurses' burnout experiences at both the levels of the nursing unit and the hospital. However, no individual-level factors were included as potential explanatory factors.

The purpose of the study presented here is to add to this body of research by investigating associations between nurse work practice environment measured at department level and individual level work-family conflict on burnout, measured as emotional exhaustion, depersonalisation and personal accomplishment among Swedish RNs.

## Study Population and Methods

### Ethics statement

The study was approved by the relevant Research Ethics committee (Regionala etikprövningsnämnden i Stockholm: Dnr 2009/1587-31/5). The participants received written information about the study, and in accordance with Swedish regulation and practice, responding to and returning the survey indicated informed consent.

### Study population and design

The present data comes from the Swedish portion of RN4CAST, a 15-nation EU 7th Framework project, based on a survey focusing on RNs working in surgical and medical inpatient care. These data have a multilevel structure with nurses nested within departments and departments nested within hospitals. Due to the nature of the data collection in the Swedish project component, no data on lower organizational levels than that of department is available. Details on the overall survey design can be found elsewhere [27]. In Sweden, RNs were approached through hospitals via the member register of the Swedish Association of Health Professionals (covering >80% of all clinically-active RNs). All RNs working in medical and surgical departments were selected (N = 33,083). The survey questionnaire was distributed by post in February 2010 through Statistics Sweden administration. At the end of the data collection period 23,087 surveys were returned (response rate about 70%, internal attrition 2–3% per item). Those RNs who responded, but did not meet the inclusion criteria (e.g. not working in medical-surgical adult in-patient care, or working in intensive care units) have been excluded from the final database. Thus, the available Swedish database consists of self-reported survey data from 11,015 RNs working with direct in-patient medical/surgical care from all acute care hospitals in Sweden. For these analyses, we omitted all departments with fewer than 10 respondents and all hospitals with fewer than 3 departments to get correct group-level variance estimates from the multilevel model [28], giving a final analytic sample of 8,620 RNs from 369 departments in 53 hospitals.

### Nurse-level and department-level variables

The individual-level variables included were: age, sex, baccalaureate degree in nursing (yes/no), years of work experience as RN and work-family conflict. Sex and year of birth were determined by linkage to the Swedish Association of Health Professionals' registry. Information on baccalaureate degree in nursing and years of work experience as RN were derived from survey items. Work-family conflict was assessed by responses to one question: ‘To what extent do you feel that your work affects your private life in a negative way?’ Response alternatives ranged from 1 = “to a very great degree”, through 5 = “to a very small degree”. For further analyses, work-family conflict was categorized into low (to a very small/small degree), medium (partly) and high (to a very high/high degree).

Work environment on department level was measured by the Practice Environment Scale of the Nursing Work Index (PES-NWI), a validated and commonly-used tool for investigating the nurse practice environment [3]. We used three sub-scales of the PES-NWI here: (1) *“Staff adequacy”* measures nurses' evaluation of the adequacy of resources to meet demands (score range: 4–16, Cronbach's α = 0.78), (2) *“Leadership & Support for RNs”* assesses key elements of leadership (score range: 4–16; Cronbach's α = 0.76), and (3) *“Nurse-physician relationship”* assesses the quality of working relations between doctors and nurses (score range:7–28; Cronbach's α = 0.89). Two sub-scales of the PES-NWI, i.e. *“Nurse impact on hospital affairs”* and *“Nursing care model”*, were not included in the analyses as those showed high multicollinarity with the other dimensions. The highest correlations were found between “Nurse impact on hospital affairs” and “Nursing care model” (r = .785), “Nurse impact on hospital affairs” and “Leadership & Support for RNs”(r = .734) and “Nursing care model” and “Leadership & Support for RNs” (r = .719). No correlations among the included dimensions exceeded 0.508. Item responses use a 4-point scale ranging from ‘strongly disagree’ to ‘strongly agree’. For information on the aggregation of sub-scales at the department level see Leineweber et al. 2014 [29].

### Outcome: Three dimensions of burnout

The outcome variables in the present analysis were measured by means of the translated and validated Swedish version of the 22-item Maslach Burnout Inventory Human Service Survey [5]. Respondents were asked to indicate their agreement with a series of questions on a seven-point rating scale ranging from 0 =  ‘never’ to 6 =  ‘every day’. In the present analysis we used all three dimensions of the scale i.e. emotional exhaustion (nine items), depersonalization (five items), and personal accomplishment (eight items). To facilitate the comparison of the dimension means, all scales were re-calculated to a 0–100 scale. Degrees of burnout experienced were calculated separately for the three dimensions and dichotomized according to the cut-off points suggested by Maslach et al. [5] into low-moderate versus high. The cut-offs applied to define cases were for emotional exhaustion ≥ 50.0, for depersonalization ≥ 44.8 and for personal accomplishment ≤ 64.6. Dichotomization of the burnout dimensions allowed application of a binary logistic modelling.

### Statistical analysis

Multilevel modelling has become a tool for explaining various sources of variation at different levels of organisation in health service research [26], [30]. A two-level logistic random intercept model with RNs nested within departments was applied to explore the variability explained by individual and department level variables taking the correlated structure of data into account [31]. We did not have variables at the department level, but the five dimensions of the PES-NWI were based on scores aggregated at the department level. In an earlier paper including the same material [29], we could show that all summated scales met statistical criteria for aggregation, which indicates that they can be considered as organisational level variables. Let *Y_ij_* denote the binary response for the *i^th^* nurse in the *j^th^* department, with *Y_ij_* = 1, if the nurse experienced any of the burnout dimension. If *π_ij_* = *P*(*Y_ij_* = 1), then the two-level random intercept logistic model can be written as,
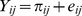
where,
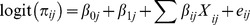
(1)

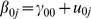
(2)

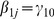
(3)


The error terms 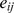 and 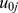
are uncorrelated such that 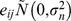 and 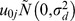, 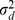 represents variation in experienced burnout between departments and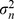, the variation among nurses within departments. This random intercept model assumed that the average probability of experiencing burnout varies randomly over departments. To make interpretations more meaningful, the nurse level variables are centered to their respective grand means [32]. Model assessment in terms of variability in experienced burnout as explained by the covariates at different levels (nurse and department) was done with reference to the empty model:
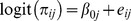
(4)


The variation in experienced burnout explained by the covariates at level one (nurse) and level two (department) was calculated as [33]:
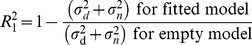
(5)

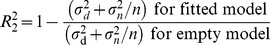
(6)where *n* is the average number of nurses in each department.

We specified three separate models: Model I adjusted for individual-level variables (i.e. age, sex, baccalaureate degree in nursing, years of experience as RN, work-family conflict), Model II adjusted for department level variables only (NWI-PES variables), and the last, Model III, fully adjusted for both individual and department level variables (NWI-PES variables). Odds ratios were calculated from the fitted models along with 95% confidence intervals. Comparisons of the competing models were done with likelihood ratio tests in the form of deviance; the smaller the value the better the model. All models were fitted using the lme4 package [34] in *R* [35].

## Results

Descriptive statistics of individual and department level variables included in the model are shown in Table 1. The mean age of participants was 41 years. The sample was highly skewed regarding sex, with males representing 6.6% of the RNs. More than half of the RNs had a baccalaureate degree in nursing and mean work experience was 11.7 years as RN. In our sample, about one third of the RNs experienced a low degree of work-family conflict, about 40% experienced a medium degree of work-family conflict, and slightly less than one quarter experienced high levels of work-family conflict. In general, RNs reported high values of personal accomplishment and lower values of emotional exhaustion and depersonalisation. However, nearly one third of the RNs (29.8%; n = 2540) were categorized as emotionally exhausted. Fewer RNs suffered from depersonalization (7.6%, n = 674) or low personal accomplishment (9.3%, n = 786). The proportion of RNs with emotional exhaustion, depersonalization or with low personal accomplishment within each department ranged from 0 to 82% (categorized as emotionally exhausted), 0 to 45% (depersonalization) and 0 to 33% (low personal accomplishment), thus indicating large variability between departments in regard to the three burnout dimensions.

**Table 1:** Descriptive statistics of outcomes, nurse-level & department-level variables.

Variables	%	Mean	SD	Min, Max	Percentiles	
					25^th^	75^th^
*Nurse level variables*						
Age (years)	.	41.0	11.1	22, 67	31.0	49.0
Male	6.6	.	.	.	.	.
Baccalaureate degree in nursing	58.7	.	.	.	.	.
Career experience as RN (years)	.	11.7	10.5	0, 43	4.0	17.0
Work-family conflict experience						
Low	35.8	.	.	.	.	.
Medium	38.4	.	.	.	.	.
High	23.8	.	.	.	.	.
*Department level variables (aggregated)*						
Staff adequacy [range: 4–16]	.	9.3	1.3	5.7, 12.6	8.4	10.2
Leadership & support for nurses [range: 4–16]	.	10.9	1.1	7.3, 13.8	10.2	11.5
Nurse-physician relationship [range: 7–28]	.	20.6	1.4	15.4, 25.3	19.8	21.5
*Dimensions of burnout*						
Emotional exhaustion	.	29.3	19.2	0, 100	24.1	51.8
Depersonalization	.	15.3	16.5	0, 100	3.4	20.7
Personal accomplishment		82.9	12.0	0, 100	75.0	91.7

*Note:* SD  =  Standard deviation; Min  =  Minimum; Max  =  Maximum.

Tables 2 to 4 show the results of the fitted models in terms of odds ratios in order to explore the relationships between the nurse level variables, the three work environment scales at the department level, and the three burnout scales. The included background variables were not related to any burnout dimension in either model. In the model adjusted only for RNs' characteristics, high work-family conflict increased the risk for emotional exhaustion, but for neither depersonalization nor personal accomplishment. These relationships changed only marginally when adjusting for department level variables (Model III). In Model II (adjusted only for department level variables), adequate staffing and good leadership and support for nurses reduced the risk for emotional exhaustion and depersonalization, whereas a good nurse-physician relationship did not show this effect. Personal accomplishment was statistically significantly related to staff adequacy, but showed no relationship with either “leadership or support for nurses” or “nurse-physician relationship”. These relationships did not change notably in the fully adjusted Model III.

**Table 2:** Two-level random intercept models of the dimension of emotional exhaustion.

	Model-I			Model-II			Model-III		
	OR	95%CI		OR	95%CI		OR	95%CI	
*Emotional exhaustion*									
Nurse-level									
Age (in years)	0.999	[0.991; 1.006]		.	.		0.997	[0.990; 1.005]	
Male	0.905	[0.739; 1.108]		.	.		0.909	[0.743; 1.113]	
Degree in nursing	1.067	[0.942; 1.209]		.	.		1.065	[0.940; 1.206]	
Career experience as RN (in years)	1.001	[0.992; 1.009]		.	.		1.001	[0.993; 1.010]	
Low work-family conflict	Ref	.		.	.		Ref	.	
Medium work-family conflict	1.076	[0.959; 1.208]		.	.		1.044	[0.929; 1.172]	
High work-family conflict	1.306**	[1.148; 1.485]		.	.		1.232*	[1.083; 1.403]	
Department-level									
Staff adequacy	.	.		0.724**	[0.684; 0.766]		0.733**	[0.693; 0.775]	
Leadership & support for nurse	.	.		0.932*	[0.872; 0.995]		0.930*	[0.871; 0.993]	
Nurse-physician relationship	.	.		0.975	[0.933; 1.019]		0.975	[0.934; 1.019]	
*Deviance of model fit*	9780			10007			9614		

*Note:* OR  =  Odds ratios; CI  =  Confidence interval; *p<0.05; **p<0.001.

**Table 3:** Two-level random intercept models of the dimension of depersonalisation.

	Model-I			Model-II			Model-III		
	OR	95%CI		OR	95%CI		OR	95%CI	
*Depersonalisation*									
Nurse-level									
Age (in years)	1.005	[0.992; 1.018]		.	.		1.003	[0.991; 1.016]	
Male	1.014	[0.733; 1.404]		.	.		1.022	[0.738; 1.413]	
Degree in nursing	1.170	[0.945; 1.449]		.	.		1.165	[0.942; 1.443]	
Career experience as RN (in years)	0.993	[0.979; 1.008]		.	.		0.995	[0.980; 1.009]	
Low work-family conflict	Ref	.		.	.		Ref	.	
Medium work-family conflict	1.082	[0.889; 1.317]		.	.		1.031	[0.847; 1.256]	
High work-family conflict	1.222	[0.985; 1.517]		.	.		1.110	[0.892; 1.380]	
Department-level									
Staff adequacy	.	.		0.856*	[0.782; 0.937]		0.864*	[0.788; 0.948]	
Leadership & support for nurse	.	.		0.867*	[0.777; 0.966]		0.871*	[0.780; 0.972]	
Nurse-physician relationship	.	.		0.971	[0.903; 1.044]		0.967	[0.899; 1.041]	
*Deviance of model fit*	4542			4686			4507		

*Note:* OR  =  Odds ratios; CI  =  Confidence interval; *p<0.05; **p<0.001.

**Table 4:** Two-level random intercept models of the dimensions of personal accomplishment.

	Model-I			Model-II			Model-III		
	OR	95%CI		OR	95%CI		OR	95%CI	
*Lack of personal accomplishment*									
Nurse-level									
Age (in years)	1.005	[0.993; 1.016]		.	.		1.003	[0.992; 1.015]	
Male	0.958	[0.701; 1.308]		.	.		0.963	[0.706; 1.314]	
Degree in nursing	0.959	[0.792; 1.161]		.	.		0.954	[0.789; 1.155]	
Career experience as RN (in years)	0.995	[0.982; 1.009]		.	.		0.997	[0.984; 1.010]	
Low work-family conflict	Ref	-		.	.		Ref	-	
Medium work-family conflict	1.038	[0.869; 1.241]		.	.		1.002	[0.838; 1.198]	
High work-family conflict	1.185	[0.972; 1.445]		.	.		1.097	[0.897; 1.341]	
Department-level									
Staff adequacy	.	.		0.883**	[0.822; 0.950]		0.888*	[0.824; 0.957]	
Leadership & support for nurse	.	.		0.979	[0.896; 1.070]		0.983	[0.897; 1.076]	
Nurse-physician relationship	.	.		0.963	[0.909; 1.021]		0.960	[0.904; 1.018]	
*Deviance of model fit*	5023			5209			5005		

*Note:* OR  =  Odds ratios; CI  =  Confidence interval; *p<0.05; **p<0.001.

The deviance statistics suggested that for all the three dimensions of burnout, Model III is most plausible in explaining the relationships between nurse level variables, department level variables, and the three outcomes, as indicated by the smallest values of deviance statistics. From Model III, the magnitude of the differential in emotional exhaustion explained by the nurse and department level variables included are 0.55% and 47% respectively; whereas for depersonalization the corresponding figures are 0.42% and 19% and for personal accomplishment 0.09% and 14%. These indicate the importance of organisational level variables in explaining the variability in the burnout dimensions among the RNs in this study.

## Discussion

In this paper we studied the associations between nurse practice environment dimensions, work-family conflict, and the different dimensions of burnout using a multilevel approach. Our final model showed a relationship between work-family conflict and emotional exhaustion, but no relationship between work-family conflict and depersonalization or personal accomplishment was found. The pronounced effect of emotional exhaustion is well in line with its' conceptualization as the most central aspect of burnout. Toppinen-Tanner have proposed that emotional exhaustion is the first symptom of burnout to develop, which may be one explanation for this [36]. Another possible explanation for the pronounced relationship with emotional exhaustion in contrast to the other two burnout dimensions could be that especially emotional and quantitative job demands have increased among RNs during the past years [37] which leads to a feeling of exhaustion.

At the department level we identified adequate staffing and good leadership and support for nurses to be related to emotional exhaustion and depersonalization. Furthermore, adequate staffing showed a positive relationship with personal accomplishment. The results of our study can be compared to results published by Li et al. [26], which are also based on the RN4CAST data from several other countries, but excluding Sweden. Li et al. investigated the impact of ‘leadership and support for nurses’, ‘nurse-physician relationship’ and ‘promotion of care quality’ on burnout among nurses taking four hierarchical levels (nurse, nursing unit, hospital, country) into account. In contrast to our findings, Li et al. found that the nurse-physician relationship affected all burnout dimensions, including personal accomplishment. However, the effect of the nursing unit level variability for the dimension of ‘leadership and support for nurses’ was only present for emotional exhaustion. As in our paper, they found the most pronounced effects for emotional exhaustion, while personal accomplishment showed the weakest effects. Although unlike Li et al., we were not able to investigate four hierarchical levels, we were able to investigate the relationship between nurse practice environment and burnout dimensions while taking individual differences and work-family conflict into account. To our knowledge our data is the first to apply a multilevel approach by taking contemporaneous work-family conflict into account.

The relationship between work-family conflict and burnout has been well researched previously [18], [38]. This study thus further reinforces previous findings regarding the (non-) relationship between work-family conflict on one hand, and emotional exhaustion and personal accomplishment on the other. Other studies have found work-family conflict to be related to emotional exhaustion and depersonalization [18], [39], but not to personal accomplishment. Thus our study deviates from previous research in regard to the relationship with depersonalization. A meta-analysis recently concluded that personal accomplishment is the weakest of the three burnout constructs [15], whereas Schaufeli et al. [40] have previously suggested the reduced personal accomplishment may be a separate construct that develops separately from emotional exhaustion and cynicism (i.e. depersonalization). Another interpretation is that personal accomplishment may well be part of the burnout construct, but individuals do not necessarily value personal accomplishments at work as much as they value other aspects such as energy and outlook on the job [15].

A large body of nurse research studying burnout has focused solely on the emotional exhaustion dimension of the syndrome, although the significance of the three-dimensional burnout model has been emphasized repeatedly [41]. In this article, we investigated all three burnout dimensions, i.e. emotional exhaustion, depersonalization and personal accomplishment as separately constructs and not as a combined measure. One might argue for the use of a unidimensional approach, where all three dimensions are combined [41]. However, several factors supported our decision to study the three dimensions separately. First, combining the dimensions might have resulted in a considerable loss of information. Second, the dimensions have been constructed in such a way that they are maximally independent from one other [5]. Third, research has shown that different variables seem to have different impacts on the different dimensions of burnout [15], [42]. Finally, given that variables such as job demands are more strongly associated with emotional exhaustion than with the other two burnout dimensions [43]–[45], we hypothesized that the three dimensions would be influenced differently by work-family conflict.

Information on the hospital level was relatively limited (hospital size, university hospital status, hospital in high/low population density areas) and none of the included hospital level variables showed a statistically significant relationship with any of the dimensions of burnout studied in this paper. A separate model incorporating hospital level variables indicated that only about 2.7%, 0.001% and 0.11% of the variability in emotional exhaustion, depersonalization and personal accomplishment, respectively, was between hospitals.

While this article contributes significant new knowledge, it is not free from limitations. Due to the cross-sectional nature of the study, no conclusions can be drawn regarding the direction of the relationships. Most research has suggested an effect from work-family conflict to burnout, i.e. increased work-family conflict increases the risk for burnout. But a reverse relationship with exhaustion increasing the risk for subsequent work-family conflict has also been found [38]. The role of work-family conflict in the development of burnout has been investigated in several studies. It has been suggested to mediate the relationship between job demands and burnout. Geurts et al. 's study [46] identified work-family interference as a mediator between work characteristics and burnout —defined there as emotional exhaustion and depersonalization — and other studies [47], [48] found that work-family conflict served as a partial mediator between job demands and job burnout. Linzer et al. [49] suggested that work-home interference had a direct as well as an indirect mediating effect on burnout. This possibly mediating effect of work-family conflict could not be addressed in our study.

In addition, the level of the department might be too rough to catch important variation in the work-environment, as many important decisions for RNs' work environment, e.g. detailed work organisation, scheduling of shift rotation, and support from the first-line manager may be determined at a lower level, e.g. that of the nursing unit or smaller work group. Future research should therefore also study the influence of lower level organisational factors as well as investigate factors in private life, e.g. family composition and working hours; variables which were not available in the current study. Third, the multicollinearity that we have identified could be due to high correlation among specific items of different dimensions, rather than among the dimensions as a whole.

In conclusion the findings suggest that adequate staffing and leadership and support for nurses are crucial for RNs' mental health. Increased nurse staffing levels in hospitals may require increases in hospital funding. Currently the reality for many hospitals in Europe, including Sweden, is a different one, with many hospitals struggling with reduced levels of funding. Demands on leadership are particularly high in times of economic difficulties and good leadership can make a difference for stressed nurses. In line with Gilleta et al [50] we emphasize the importance of clarifying admiration, respect, and trust of staff to heighten awareness of collective interest and support staff in achieving collective goals. Our findings also highlight the importance of hospital managers developing policies and practices to facilitate the successful combination of work with private life for employees. Supervisors should model effective work–family management strategies, and provide information and advice which can reduce the extent to which work interferes with family responsibilities [51], [52].
